# HIV-1 Entry and *Trans*-Infection of Astrocytes Involves CD81 Vesicles

**DOI:** 10.1371/journal.pone.0090620

**Published:** 2014-02-28

**Authors:** Lachlan R. Gray, Stuart G. Turville, Tina L. HItchen, Wan-Jung Cheng, Anne M. Ellett, Hamid Salimi, Michael J. Roche, Steve L. Wesselingh, Paul R. Gorry, Melissa J. Churchill

**Affiliations:** 1 Centre for Biomedical Research, Burnet Institute, Melbourne, Victoria, Australia; 2 Department of Biochemistry and Molecular Biology, Monash University, Victoria, Australia; 3 The Kirby Institute, Darlinghurst, New South Wales, Australia; 4 South Australian Health and Medical Research Institute, Adelaide, South Australia, Australia; 5 Department of Microbiology and Immunology, University of Melbourne, Melbourne, Victoria, Australia; 6 Department of Infectious Diseases, Monash University, Victoria, Australia; 7 Department of Microbiology, Monash University, Victoria, Australia; 8 Department of Medicine, Monash University, Victoria, Australia; George Mason University, United States of America

## Abstract

Astrocytes are extensively infected with HIV-1 *in vivo* and play a significant role in the development of HIV-1-associated neurocognitive disorders. Despite their extensive infection, little is known about how astrocytes become infected, since they lack cell surface CD4 expression. In the present study, we investigated the fate of HIV-1 upon infection of astrocytes. Astrocytes were found to bind and harbor virus followed by biphasic decay, with HIV-1 detectable out to 72 hours. HIV-1 was observed to associate with CD81-lined vesicle structures. shRNA silencing of CD81 resulted in less cell-associated virus but no loss of co-localization between HIV-1 and CD81. Astrocytes supported trans-infection of HIV-1 to T-cells without *de novo* virus production, and the virus-containing compartment required 37°C to form, and was trypsin-resistant. The CD81 compartment observed herein, has been shown in other cell types to be a relatively protective compartment. Within astrocytes, this compartment may be actively involved in virus entry and/or spread. The ability of astrocytes to transfer virus, without *de novo* viral synthesis suggests they are capable of sequestering and protecting virus and thus, they could potentially facilitate viral dissemination in the CNS.

## Introduction

Human immunodeficiency virus type 1 (HIV-1) penetrates the central nervous system (CNS) during acute infection [1]. Progression of HIV-1 disease within the CNS frequently causes HIV-1 encephalitis (HIVE), HIV-associated dementia (HAD) or less severe HIV-associated neurocognitive disorders (HAND) [2], collectively affecting approximately 50% of the infected population [3], [4]. Within the CNS, HIV-1 productively infects resident perivascular macrophages and microglia [5], [6]. In contrast, astrocytes undergo a restricted infection and produce little or no virus [7]–[9]. Whilst astrocyte infection is restricted, their infection leads to cellular dysfunction, resulting in altered gene expression, loss of neuronal support, dysregulation of glutamate levels, and altered blood-brain barrier integrity, all of which contribute to HAND [2], [8], [10], [11].

Infected astrocytes have been shown to express detectable levels of early, multiply spliced HIV-1 gene products, including *nef* [7], [12], [13]. Thus astrocyte infection is restricted whereby multiply spliced HIV-1 mRNA may be selectively expressed without completion of the virus replication cycle. Generally, it is believed that astrocyte infection is controlled by two phases of restriction; the acute phase and the dormant phase. During the acute phase, replication in astrocytes results in low-level virus production, which is controlled post-transcriptionally [14], [15]. During the dormant phase, there is restricted expression of viral transcripts caused by low-level basal long terminal repeat (LTR) promoter activity, which can be overcome with cytokine/chemical stimulation [16], [17]. The dormant phase is also likely to represent long-term or latently infected cells, which are a current barrier to HIV-1 eradication efforts. These two phases of the restricted state result in initial suppression of virion production despite high level mRNA synthesis, followed by eventual suppression of mRNA transcription [8].

Several studies have examined the molecular mechanisms involved in the restriction of HIV-1 production in astrocytes and revealed that virus replication is restricted at multiple steps within the virus lifecycle [7]–[9]. Astrocytes lack the CD4 receptor, which is required for classical HIV-1 entry, and infection is thought to be both CD4 and coreceptor independent [18], [19]. Viral entry alone is thought to be a significant bottleneck to fully productive infection in astrocytes, as studies psuedotyping HIV with envelopes from vesicular stomatitis virus (VSV) or murine leukemia virus (MLV) achieved productive infection in astrocytes [20], [21]. Other studies have identified a cellular block in Rev function impairing nucleocytoplasmic transport of Rev-dependent HIV-1 mRNA [22], [23], translational blocks despite high mRNA levels [14], [15], and a heightened protein kinase R (PKR)-mediated antiviral response due to low levels of the PKR inhibitor, TAR-RNA binding protein (TRBP) [8], [24].

Recovery of infectious virus from astrocytes has been demonstrated using several approaches, including stimulation with proinflammatory cytokines TNFα and IL1-β, or when co-cultured with CD4+ cells [18], [25], [26]. These studies provide evidence that given the appropriate stimuli *in vivo*, astrocytes have the potential to act as a source of *de novo* HIV-1 within the CNS. The frequency of astrocyte infection was previously believed to be 3% [5], [7], [27], but more recent work in our laboratory using highly sensitive techniques has indicated that this can be as high as 19% [28]. This relatively high infection frequency coupled with the fact that astrocytes are the most abundant cell type in the brain (approximately 0.4 – 2.0 × 10^12^ cells), numerically suggests they may represent a significant HIV-1 reservoir within the CNS [7], [29]. Additionally, the immune privileged nature of the CNS and the reduced bioavailability [30] and activity of antiviral drugs [31] add further complexity to efforts aimed at controlling and preventing HIV-1 infection within the brain.

Here we aimed to characterise HIV-1 entry into astrocytes to elucidate the entry pathway and the cellular compartments that are involved in infection of astrocytes. Furthermore, we analysed the ability of astrocytes to support *trans*-infection and identify the compartment responsible for this form of viral dissemination. We used novel immunofluorescence techniques to address these questions using replication competent cell free HIV-1 with relevant HIV-1 envelope glycoproteins. Consistent with previous studies, we observed uptake of HIV-1 into vesicle compartments [32], [33] and we further show that these compartments are lined with CD81. We also demonstrate that HIV-1 can be subsequently released and transmitted to CD4+ T-cells without *de novo* synthesis, suggesting astrocytes support *trans*-infection. The results of our study suggest that the CD81 compartment can harbor and protect HIV-1 whilst also acting as a vehicle to facilitate *trans*-infection of neighboring cells. This pathway may potentially have a role in HIV-1 dissemination within the brain.

## Materials and Methods

### Cell lines and primary cells

The SVG astrocyte cell line [34] was cultured in Minimum Essential Medium (MEM) (Life Technologies, NY, USA) supplemented with 20% (vol/vol) heat-inactivated fetal calf serum (HI-FCS), 100 µg/ml of penicillin and streptomycin, and 2 mM of GlutaMAX (Life Technologies). The 293T cell line (ATCC) was cultured in Dulbecco's Modified Eagle Medium (DMEM) (Life Technologies) supplemented with 10% (vol/vol) HI-FCS, 100 µg/ml of penicillin and streptomycin, and 2 mM of GlutaMAX. The JLTRG cell line [35] was cultured in Roswell Park Memorial Institute (RPMI) media (Life Technologies) supplemented with 10% (vol/vol) HI-FCS, 100 µg/ml of penicillin and streptomycin, and 2 mM of GlutaMAX.

### Production and quantitation of virus stocks

HIV-1 BaL was produced by transfection of 293T cells with the provirus expression plasmid pBaL (HIV-1 subtype B molecular clone). The supernatants containing virus were harvested 48 h later, by ultracentrifugation over a 20% (wt/vol) sucrose cushion at 120,000 × g for 1 h, quantified by p24 ELISA, and stored at -80°C. EGFP content-labelled HIV-1 YU2_ciGFP_ was produced by co-transfection of 293T cells with the provirus expression plasmid pYU2 (HIV-1 brain-derived molecular clone) and pTI3 at a ratio of 15∶1. pTI3 encodes for HIV-1 GAG-iGFP and was constructed by replacing the EGFP open reading frame in peGFP-N1 (Clontech, CA, USA) with HIV iGFP [36], [37]. Co-expression leads to incorporation of HIV Gag-iGFP in *trans* and latter cleavage of EGFP from HIV Gag during viral maturation. The supernatants containing virus were harvested 48 h later, filtered through 0.45 µm filters, and stored at −80°C.

### HIV-1 p24 ELISA

Viral stocks and cell-associated virus was quantified using the HIV-1 p24^CA^ antigen capture assay kit (SAIC Frederick, AIDS Vaccine Program, MD, USA), according to the manufacturer's protocol.

### Virus half-life assays

SVG cells were seeded at 5,000 cells/well in 96-well plates. The following day, SVG cells were pulsed with non-saturating amounts (1.0 µg/ml p24, as determined via escalating dose loading and saturation curve) of HIV-1 BaL for 2 h at 37°C. Cells were then washed extensively and virus half-life was determined by HIV-1 p24 ELISA over a 72 h period.

### *trans*-infection assays

We define *trans*-infection as the uptake and short-term transfer of HIV-1 to permissive cells as outlined previously in HIV-1 exposed dendritic cells [38]. To observed transfer in the short-tem, independent of *de* novo infection, we performed transfer experiments as described previously. Briefly, SVG cells were loaded with virus as detailed above, either at 4°C or 37°C [38]. After virus loading, some samples were treated with 0.05% TrypLE (Life Technologies) at 37°C for 10 mins to remove residual attached surface accessible virus. Following washing, cells were co-cultured with 10,000 cells/well of JLTRG cells overnight (16 h). The following day the JLTRG cells were transferred to new plates and cultured for a further 5 days before analysing EGFP expression via FACS. Media treated SVG cells were included as a negative control.

### Immunofluorescence assays

To synchronise viral entry events, SVG cells were spinoculated at 4°C for 1 h with the EGFP content-labelled HIV-1 YU2_ciGFP_, followed by extensive washing and fixation with 4% paraformaldehyde (1 × PBS) at 0, 15, 45 and 135 mins post-infection. Samples were immunofluorescently stained for vesicle and endosomal markers using mouse anti-human antibodies specific for CD81, EEA1, CD63, CD107b and isotype control (JS-81, 14/EEA1, H5C6, H4B4, MOPC-21, clones respectively) (Becton Dickinson, NJ, USA) at 1∶200 and goat-anti mouse Alexa Fluor 555 (Life Technologies) at 1∶400. Nuclei were counterstained using Hoechst 33258 (Life Technologies). Samples were imaged on a Zeiss Cell Observer microscope using an air objective (40×, NA 0.75). IMARIS software (Bitplane, Zurich, Switzerland) was used to analyse images and quantify co-localization using the Coloc module as previously described [39]. Multiple fields of view and three independent experiments were used to generate the data.

### shRNA knockdown of CD81

SVG cells were seeded at 30,000 cells/well in a 12-well plate. The following day the media was replaced with full media supplemented with 0.5 µg/ml polybrene and cells were transduced with 20 μl of shRNA lentiviral particles specific for either CD81 or negative scrambled shRNA (Santa Cruz Biotechnology, TX, USA). 24 h post-transduction the media was changed and 48 h post-transduction, puromycin was introduced in escalating doses. A week later cells were cultured in 2 µg/ml puromycin and cells were analysed via FACS for expression of CD81 using a FITC-conjugated mouse anti-human antibody specific for CD81 (Becton Dickinson).

## Results

### Astrocytes harbor short-term HIV-1 viral reservoirs

**Figure 1:**
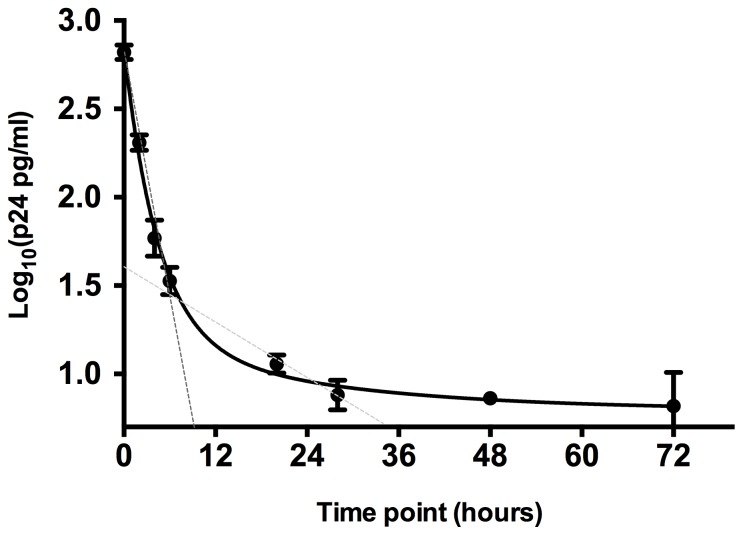
Astrocytes harbor short-term HIV-1 viral reservoirs. Virus half-life assays were performed using HIV-1 BaL and the immortalised human foetal astrocyte cell line, SVG. Cell-associated HIV-1 p24 was quantified via ELISA over a time course from 0 to 72 h. The curve of best fit is shown in black. The initial virus half-life of 1.2 h is shown via a dotted dark gray line and the slower rate of 9.5 h is shown via a dotted light gray line. Dot points and error bars denote the mean and standard deviation, respectively. Data is representative of three independent experiments.

We first tested the ability of astrocytes to bind and harbor HIV-1 over a time course to determine if they were capable of supporting the establishment of short-term viral reservoirs. Non-saturating amounts of HIV-1 BaL were used to load astrocytes, followed by extensive washing and analysis of the cell-associated HIV-1 p24. Astrocytes demonstrated a biphasic decay of virus, with an initial half-life of 1.2 hours followed by a slower rate of 9.5 hours (Figure 1). Cell associated virus was detectable out to 72 hours, potentially suggesting they are capable of supporting the establishment of short- to mid-term viral reservoirs.

### HIV-1 associates with CD81-lined compartments in astrocytes

**Figure 2:**
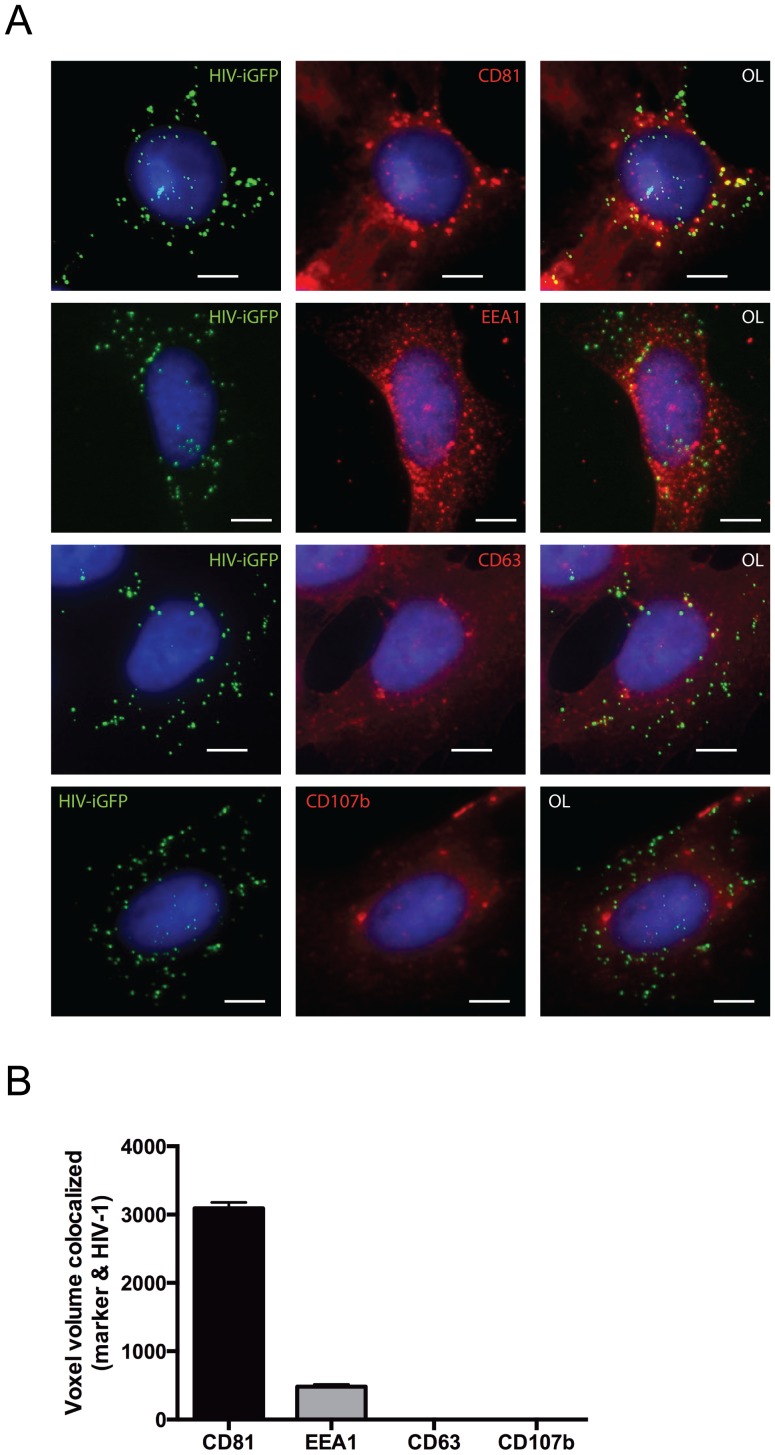
HIV-1 colocalizes with CD81-lined vesicle compartments in astrocytes. (A) SVG astrocyte cells were infected with an EGFP content-labelled HIV-1 YU2 virus (green) and samples were harvested at 135 mins post-infection. Cells were stained with the vesicle/endosomal markers CD81, EEA1, CD63 or CD107b (red) and nuclei counterstained with DAPI (blue). Panels represent the individual fluorescent channels with the overlay (OL) on the far right. Scale bars are 10 μm. Images are representative of multiple fields of view and three independent experiments. (B) Quantification of colocalization of vesicle/endosomal markers with HIV-1 using IMARIS software. Bar graphs and error bars represent the mean and standard deviation, respectively. Data are a compilation of multiple fields of view and three independent experiments.

To determine the cellular compartment involved in sequestering HIV-1 in astrocytes, we next performed immunofluorescence studies. Astrocytes were infected with an EGFP content-labelled HIV-1 and were immunofluorescently stained for vesicle and endosomal markers including CD81, EEA1, CD63 and CD107b. HIV-1 was found to colocalize with the vesicle marker CD81 (Figure 2A), with colocalization quantified using IMARIS image software (Figure 2B). This colocalization increased overtime and was most pronounced at the 135-minute time point. In contrast, the endosomal and lysosomal markers EEA1, CD63 and CD107b had minimal or no colocalization with HIV-1. These findings suggest that HIV-1 may use CD81-lined vesicles as a potential short-term reservoir compartment. Additionally, this compartment may also be responsible as the entry site of HIV-1 into astrocytes.

### Reducing CD81 expression did not alter the association between HIV-1 and CD81-lined compartments

**Figure 3:**
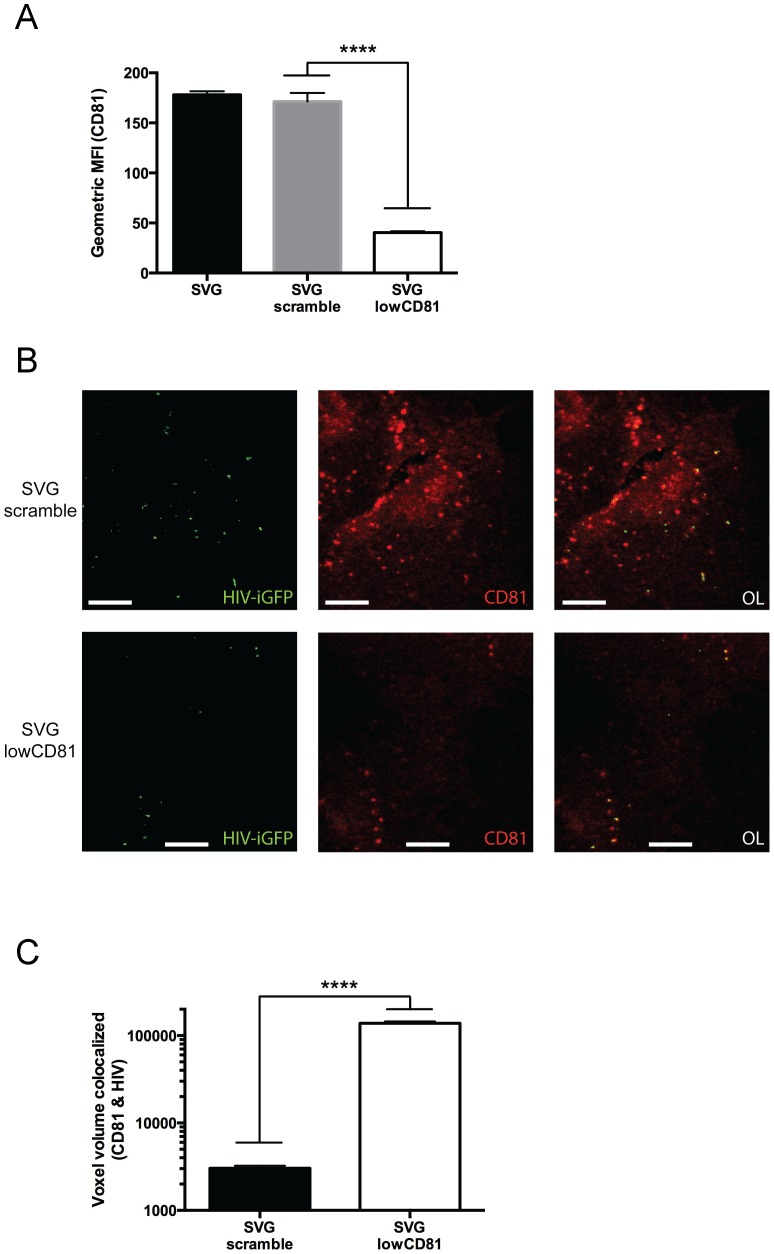
CD81-HIV-1 association is maintained when CD81 expression is reduced. (A) CD81 expression levels of the parental SVG cell line, SVG-scramble (cells treated with shRNA-scramble) and SVG-lowCD81 (cells treated with shRNA-CD81) cells as determined via FACS analysis. The data are expressed as mean values and error bars represent standard deviation. (B) SVG-scramble and SVG-CD81 cells were infected with an EGFP content-labelled HIV-1 YU2 virus (green) and samples were harvested at 135 mins post-infection. Cells were stained with the vesicle marker CD81 (red). Panels represent the individual fluorescent channels with the overlay (OL) on the far right. Scale bars are 10 μm. Images are representative of multiple fields of view and three independent experiments. (C) Quantification of colocalization of CD81 with HIV-1 using IMARIS software. The data are expressed as mean values and error bars represent standard deviation. Data are a compilation of multiple fields of view and three independent experiments. *P* values were calculated using a parametric unpaired t test; ****, *P* < 0.0001.

To determine whether CD81 was directly involved in recruiting HIV-1 to CD81-lined compartments, we next performed shRNA studies targeting CD81. Astrocytes were treated with lentiviral particles encoding for shRNA specific for CD81 (SVG-lowCD81) or a non-specific scrambled shRNA control (SVG-scramble). CD81 levels were significantly silenced by 77% (*P*<0.0001) using shRNA specific for CD81 (Figure 3A). In contrast, the scrambled shRNA did not alter CD81 levels. We next repeated the virus loading and immunofluorescence studies done previously using these two new cell lines. The SVG-lowCD81 cells maintained their association between CD81-lined compartments and HIV-1, despite reduced CD81 levels (Figure 3B). The amount of CD81-HIV-1 colocalization was significantly higher (*P*<0.0001) in the SVG-lowCD81 cells compared to SVG-scramble cells (Figure 3C). These results suggest that CD81 acts as a marker of the vesicle compartment in which HIV-1 localizes, but may not have a direct role in recruitment of virus to this compartment.

### Astrocytes support *trans*-infection of HIV-1

**Figure 4:**
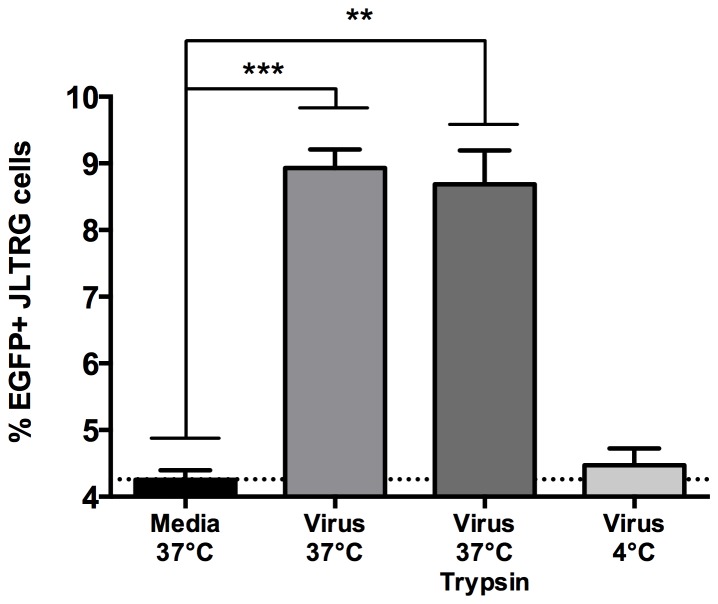
Astrocytes are capable of *trans*-infection. FACS analysis of EGFP expression in JLTRG cells following co-culture with SVG cells loaded with virus under different conditions (37°C, 37°C followed by trypsin treatment, or 4°C). Media treated cells were included as a negative control for background fluorescence in the JLTRG cells. The data are expressed as mean values and error bars represent standard deviation. Data are a compilation of three independent experiments. *P* values were calculated using a parametric unpaired t test; ***, *P* = 0.0005; **, *P* = 0.001.

To test the hypothesis that astrocytes can support *trans*-infection we performed virus loading and transfer experiments to T-cells. We define *trans*-infection as the uptake and short-term transfer of HIV-1 to permissive cells in the absence of *de novo* infection. Astrocytes were loaded with non-saturating amounts of HIV-1 at 37°C alone, 37°C followed by mild trypsin treatment, or 4°C. Media treated cells were included as a negative control. Following virus loading and extensive washing, cells were co-cultured with the JLTRG reporter T-cell line. Transfer of virus from astrocytes to T-cells results in their infection and subsequent EGFP expression was measured using FACS. Compared to media treated astrocytes, cells loaded with virus at 37°C or 37°C + trypsin resulted in a significant induction of EGFP expression in T-cells (*P* = 0.0005, *P* = 0.001, respectively) (Figure 4). In contrast, astrocytes loaded with virus at 4°C resulted in no significant increase in EGFP in T-cells compared to media treated astrocytes. These results suggest that astrocytes are capable of supporting *trans*-infection of HIV-1 with subsequent transfer to T-cells. In addition, the virus-containing compartment required 37°C to form and was insensitive to trypsin treatment suggesting these structures were internal to the cell and may assist in protecting HIV-1.

## Discussion

The aim of this study was to identify the entry pathway of HIV-1 into astrocytes and to determine whether astrocytes were capable of supporting *trans*-infection. In astrocytes we demonstrated that cell-associated HIV-1 undergoes biphasic decay and could be harbored for long periods of time. We identified that HIV-1 was taken up into vesicle compartments lined with CD81 and that alteration of CD81 levels did not influence the colocalization of HIV-1 and CD81. Finally, we revealed that astrocytes are capable of supporting *trans*-infection. Together these findings shed new light on the entry process of HIV-1 into astrocytes and suggest they may also play an active role in viral dissemination within the CNS.

The viral kinetics of HIV-1 infection of astrocytes suggests that these cells may harbor virus for long periods of time. During the initial phase of infection the virus likely sequesters in multiple compartments, each with its own rate of decay, as evidenced by the observed biphasic half-life of the virus. The bulk of the virus associates with a rapidly decaying compartment with a half-life of 1.2 hours while the remaining virus associates with a slower decaying compartment with a half-life of 9.5 hours. This later compartment likely comprises CD81-lined vesicles, which have been shown elsewhere to be relatively protective structures in other cell types [40], [41], and is supported by our data with an increased virus half-life. Previous reports identified that HIV-1 has a very rapid decay, with a half-life in the order or minutes to hours (0.4 – 1.8 hours)[27], [42]. Together, these findings suggest that astrocytes may play a role in sequestering virus, resulting in prolonged virus viability and potentiating increased infection and spread within the CNS.

Immunofluorescence analysis of astrocytes after brief exposure to HIV-1 demonstrated predominant association with CD81 stained structures. CD81 and HIV-1 were concentrated into compact, apparently intracellular structures, with virions accumulating into several distinct foci throughout the cell. CD81-HIV-1 colocalization was also observed to increase overtime, suggesting either HIV-1 actively migrates to CD81 vesicles for protection, or that virus in other compartments is preferentially degraded while virus in CD81 vesicles is protected. The association of HIV-1 with CD81-positive compartments has been reported previously in dendritic cells [40], [41], [43] and macrophages [44], but here we show for the first time that these structures also form in astrocytes. Of particular interest, these same reports have shown that this compartment is involved in facilitating *trans*-infection of T-cells via dendritic cells. Much earlier work from our group identified that HIV-1 associates in vesicle-like structures but the exact compartment remained elusive [32]. Clarke *et al.*, also suggested these structures were most likely formed via macropinocytosis or phagocytosis [45] and would be consistent with the involvement of the mannose receptor [33]. We conclude that these vesicle-like structures can be identified using CD81, as shown here and elsewhere, and they play a crucial role in HIV-1 entry into astrocytes, whilst also supporting *trans*-infection of neighboring cells.

We also observed a weaker association between HIV-1 and the early endosome marker, EEA1. This may represent multiple entry routes of the virus into astrocytes, which is again supported by the biphasic virus half-life within astrocytes. We chose our various vesicle, endosome and lysosome markers to cover a wide spectrum of different cellular structures involved in endocytosis and cellular uptake. Of these, CD81 is a marker for vesicles, and represents the earliest form for cellular uptake. Therefore it is not unexpected that we found the strongest association with this compartment. Future work is required to determine the role each of these compartments plays in entry and *trans*-infection. It is also unclear whether these are transient events with subsequent progression into late endosome/lysosome causing degradation of their contents.

The silencing of CD81 in astrocytes resulted in a significant reduction in CD81 expressed on the cell surface. However, immunofluorescence studies performed with these same cells did not adversely affect the association between HIV-1 and CD81. We also observed a reduction in the amount of overall virus associated with the CD81 depleted cells. When colocalization was compared to the parental cells we observed an increased reliance on CD81-lined compartments in the CD81 depleted cells. Because CD81 expression was not completely eliminated, it is unclear whether CD81 itself has a direct role in recruiting HIV-1 into this compartment. Reducing CD81 levels could result in two distinct outcomes: 1) it may affect the stability of the vesicle structures resulting in fewer forming or 2) it may simply deplete CD81 content from these vesicles with no affect on total vesicle numbers. Future work will explore these two scenarios using CD81-mutant cell lines, and immunofluorescence of associated vesicle markers. Together these results suggest that CD81 serves as a marker of the cellular compartment with which HIV-1 associates in astrocytes.

*Trans*-infection experiments revealed that astrocytes are indeed capable of facilitating transfer of virus to neighboring cells in the absence of *de novo* infection. To our knowledge, this is the first demonstration that astrocytes possess this capability *in vitro*. While *trans*-infection has been definitively shown in dendritic cells [36], [40], [41], this is the first report within CNS cells, and provides new insights into the potential of astrocytes to influence viral dissemination within the brain.

In addition to the *trans*-infection studies, experiments performed at 4°C or in the presence of trypsin revealed important insights. Firstly, physiologically relevant temperatures were required for the formation of the compartment involved in *trans*-infection. This suggests that the uptake of virus is an active process and likely involves enzymes and reactions that can only function at 37°C. Secondly, the compartment harboring virus does not involve virus adhered to the outside of the cell, as trypsin treatment would effectively remove this virus. Additionally, the data also suggests that the compartment may be intracellular with no access to the extracellular space, rendering it trypsin-resistant. Cavrois *et al.*, recently performed a study in dendritic cells to demonstrate that *trans*-infection occurred primarily by surface-accessible HIV-1 and suggested that internalized HIV does not play a role in *trans*-infection [46]. This finding somewhat contradicts that of our own, but may be explained due to the different model and cell type used. More recently, Yu *et al.* concluded that Cavrois *et al.* findings were limited and successfully demonstrated that the vast majority of HIV-1 involved in *trans*-infection is derived from internalized non-endosomal compartments that remain contiguous with the plasma membrane [41]. While these findings are more corroboratory with our own data, the trypsin-resistant nature of our compartment may represent an astrocyte-specific structure.

Several groups have demonstrated that infectious HIV-1 can be recovered from astrocytes up to 5 months post-infection [47], [48]. The ability of astrocytes to harbor and transmit virus without replication may represent an important mechanism by which HIV-1 can evade the immune system and antiretroviral drugs. Additionally, this non-replicative mode of HIV-1 persistence and transmission may potentially be involved in HIV-1 entry and spread within the CNS. Furthermore, this novel virus/astrocyte interaction may also represent an additional way in which HIV-1 causes astrocyte dysfunction in the absence of viral replication. The interplay between the virus and intracellular vesicles could alter the normal astrocyte vesicle sorting events required for recycling of neurotransmitters and export of neurotropic factors. Further elucidation of non-replicative astrocyte infection is required to comprehensively understand HIV-1 entry, spread and persistence within the CNS.
