# Correction: HIV-1 Entry and *Trans*-Infection of Astrocytes Involves CD81 Vesicles

**DOI:** 10.1371/journal.pone.0093417

**Published:** 2014-03-20

**Authors:** The PLOS ONE Staff

The name of the third author is spelled incorrectly. The correct name is: Tina L. Hitchen. The correct Citation is: Gray LR, Turville SG, Hitchen TL, Cheng W-J, Ellett AM, et al. (2014) HIV-1 Entry and *Trans*-Infection of Astrocytes Involves CD81 Vesicles. PLoS ONE 9(2): e90620. doi:10.1371/journal.pone.0090620.
